# Bell’s and Dunglison’s Medical Libraries

**Published:** 1837

**Authors:** 


### Art IX.—Bell's and Dunglison's Medical Libraries.

These publications, running parallel to each, are in regular, and, as far as we can discover, healthful progress. We trust they are widely disseminated. As far as we have looked into them, the selection of European works is made with good judgment—referring to the wants of the American profession ; while the price, including the postage, is actually much lower than the same works could be obtained for, from the booksellers. Many of them, indeed, have not before been reprinted in this country.

The title of Dr. Bell's publication, the older of the two, is the "*Select Medical Library, and Eclectic Journal of Medicine,*" published by Haswell, Barrington & Haswell, Philadelphia.

That of Dr. Dunglison, the "*American Medical Library and Intelligencer,*" by Adam Waldie, of the same city. D.

